# Proteomic analysis of the biomass hydrolytic potentials of *Penicillium oxalicum* lignocellulolytic enzyme system

**DOI:** 10.1186/s13068-016-0477-2

**Published:** 2016-03-17

**Authors:** Wenxia Song, Xiaolong Han, Yuanchao Qian, Guodong Liu, Guangshan Yao, Yaohua Zhong, Yinbo Qu

**Affiliations:** State Key Laboratory of Microbial Technology, School of Life Science, Shandong University, No.27 Shanda South Road, Jinan, 250100 Shandong China; National Glycoengineering Research Center, Shandong University, No.27 Shanda South Road, Jinan, 250100 Shandong China

**Keywords:** *Penicillium oxalicum*, *Trichoderma reesei*, Cellulase, Enzymatic hydrolysis, Proteomic, Adsorption

## Abstract

**Background:**

The mining of high-performance enzyme systems is necessary to develop industrial lignocellulose bioconversion. Large amounts of cellulases and hemicellulases can be produced by *Penicillium oxalicum*. Hence, the enzyme system of this hypercellulolytic fungus should be elucidated to help design optimum enzyme systems for effective biomass hydrolysis.

**Results:**

The cellulolytic and xylanolytic activities of an SP enzyme system prepared from *P. oxalicum* JU-A10 were comparatively analyzed. Results indicated that the fungus possesses a complete cellulolytic-xylanolytic enzyme system. The cellobiohydrolase- and xylanase-specific activities of this system were higher than those of two other enzyme systems, i.e., ST from *Trichoderma reesei* SN1 and another commercial preparation Celluclast 1.5L. Delignified corncob residue (DCCR) could be hydrolyzed by SP to a greater extent than corncob residue (CCR). Beta-glucosidase (BG) supplemented in SP increased the ability of the system to hydrolyze DCCR and CCR, and resulted in a 64 % decrease in enzyme dosage with the same glucose yield. The behaviors of the enzyme components in the hydrolysis of CCR were further investigated by monitoring individual enzyme dynamics. The total protein concentrations and cellobiohydrolase (CBH), endoglucanase (EG), and filter paper activities in the supernatants significantly decreased during saccharification. These findings were more evident in SP than in the other enzyme systems. The comparative proteomic analysis of the enzyme systems revealed that both SP and ST were rich in carbohydrate-degrading enzymes and multiple non-hydrolytic proteins. A larger number of carbohydrate-binding modules 1 (CBM1) were also identified in SP than in ST. This difference might be linked to the greater adsorption to substrates and lower hydrolysis efficiency of SP enzymes than ST during lignocellulose saccharification, because CBM1 not only targets enzymes to insoluble cellulose but also leads to non-productive adsorption to lignin.

**Conclusions:**

*Penicillium oxalicum* can be applied to the biorefinery of lignocellulosic biomass. Its ability to degrade lignocellulosic substrates could be further improved by modifying its enzyme system on the basis of enzyme activity measurement and proteomic analysis. The proposed strategy may also be applied to other lignocellulolytic enzyme systems to enhance their hydrolytic performances rationally.

**Electronic supplementary material:**

The online version of this article (doi:10.1186/s13068-016-0477-2) contains supplementary material, which is available to authorized users.

## Background

Lignocellulose is the most abundant renewable biomass on earth and thus is considered as the most promising sustainable source of liquid fuels and related chemicals. As such, lignocellulose has been extensively investigated because of exacerbating energy crisis and other environmental issues [1–4]. Lignocellulose consists mainly of cellulose, hemicelluloses, and lignin. These polymers are interlinked into a hetero-matrix, and their relative abundance varies substantially on the basis of biomass type [5]. The breakdown of lignocellulosic biomass involves the formation of long-chain polysaccharides, such as cellulose and hemicelluloses, and their subsequent hydrolysis into their component five- and six-carbon chain sugars [1]. In biofuel production, these sugars can be converted to bioethanol through fermentation. Lignocellulosic biomass possesses a complex structural and chemical composition; hence, its degradation requires a broad range of enzymes [6]. Conversely, natural lignocellulolytic enzyme systems from filamentous fungi are generally deficient in terms of certain components and thus require enzyme supplementation to hydrolyze complex lignocellulosic materials completely. Therefore, the composition of these enzyme systems should be modified on the basis of biomass materials [7–9]. The composition of cellulase mixtures should also be optimized to enhance hydrolytic efficiency [10–13]. However, the design of cost-effective lignocellulolytic enzyme cocktails is limited by the lack of knowledge on whole enzyme systems and exact quantities of individual cellulolytic proteins secreted by lignocellulose-degrading microorganisms. Indeed, lignocellulolytic enzyme complexes should be optimized on the basis of proteomic analysis to develop efficient bioconversion processes.

*Penicillium oxalicum* 114-2 is a fast-growing cellulolytic fungus with good lignocellulose hydrolytic performance, and this strain was first isolated from decayed straw-covered soil in 1979 [14]. Since this strain was subjected to long-term improvement through repeated mutagenesis and screening, its cellulase productivity has remarkably increased, and its enzyme system possesses a more balanced composition than that of the widely used cellulase producer *Trichoderma reesei* [15]. The hypercellulolytic mutant *P. oxalicum* JU-A10 has been used for industrial-scale production of cellulase preparation in China since 1996 [16]. However, commercial enzyme mixtures produced by this strain at industrial scale have yet to be characterized in detail, and their proteinaceous components have yet to be elucidated. Proteomic methods have been employed to comprehensively evaluate enzymes involved in lignocellulosic biomass degradation [17, 18], would help in understanding the properties of this enzyme system.

As byproducts of xylose industry, both corncob residues (CCR) and delignified corncob residues (DCCR) contain abundant cellulose, so they are potential substrates in bioethanol field. In this study, the hydrolytic performances of three commercial cellulase preparations (SP from *P. oxalicum* JU-A10, ST from *T. reesei* SN1, and Celluclast 1.5L from Novozymes) on CCR and DCCR were compared. To understand the differences in hydrolytic performances, we analyzed the dynamics of enzyme activities and the proteome of the cellulase preparations. Our study provided comprehensive insights into the protein repertoire and their relative abundances in the secretome of the hypercellulolytic *P. oxalicum*. Our study also proposed targets to optimize enzyme systems and to facilitate the rational designing of highly effective enzyme cocktails for lignocellulose bioconversion.

## Results

### Chemical components of CCR and DCCR

Corncobs are highly abundant agricultural residues in China. As such, these residues have been used to produce xylose by being pretreated with diluted acid or xylo-oligosaccharides after they are extracted with hot water at high pressure [19]. Corncob residues (CCR) can be utilized as lignocellulose substrates to produce ethanol. Delignified corncob residues (DCCR) are obtained from CCR by treating with alkali to remove lignin, as described by Zhao et al. [20].

In the study, CCR and DCCR were milled to small particle sizes, and then the chemical components were determined [21]. The results are shown in Table 1. The CCR were mainly composed of 56.68 % glucan, 5.01 % xylan, and 23.23 % lignin. The lignin and xylan contents of DCCR, especially acid-insoluble lignin content, were lower than those of CCR. The contents of glucan, xylan, and lignin were 73.75, 3.50, and 2.38 % in DCCR, respectively.

**Table 1:** Chemical components of pretreated lignocellulosic substrates

Substrates	Glucan (%)	Lignin (%)		Xylan (%)	Ash (%)	Others (%)
		AIL	ASL			
CCR	56.68 ± 2.06	22.16 ± 0.46	1.07 ± 0.04	5.01 ± 0.28	1.53 ± 0.03	13.55
DCCR	73.75 ± 0.56	1.96 ± 0.13	0.42 ± 0.13	3.50 ± 0.68	1.96 ± 0.13	18.41

### Evaluation of the hydrolytic ability of the cellulase preparations

A complete cellulase system consists of three classes of enzymes, namely cellobiohydrolase (CBH), endoglucanase (EG), and beta-glucosidase (BG) [22]. The enzymatic process can be accomplished through the synergism of these three enzymes [23, 24]. Endoglucanase can hydrolyze glycosidic bonds in the interior of cellulose chains, whereas cellobiohydrolase acts preferentially on chain ends. The products of the enzymatic reactions are mostly disaccharides known as cellobiose and, to a lesser extent, cello-oligosaccharides, which can be further hydrolyzed by beta-glucosidase [1].

To gain insight into the predominant enzymes in SP, we determined the specific activities by comparing model substrates by comparing with ST and Celluclast 1.5L (Table 2). Although SP did not display higher beta-glucosidase and beta-xylosidase activities, the preparation showed 9.83 and 2.17 times higher specific activity on xylan than Celluclast 1.5L and ST, respectively. Qing et al. suggested that insufficient xylanase activity resulting in high xylo-oligomer concentrations in the hydrolysis broth could severely inhibit cellulase activity [25]. In other words, SP may relieve the accumulation of xylo-oligomers to obtain increased final glucose yield. In addition, the cellobiohydrolase activity, which is the key component in the large-scale bioconversion of lignocelluloses [26], was noted to be about 3.5 and 2.3 times higher in SP on *p*NPC than that of Celluclast 1.5L and ST (*T. reesei* cellulase preparations). In general, SP cellulase preparation has the potentials of biomass hydrolysis because of its complete cellulase system with various lignocellulolytic enzymes, such as cellobiohydrolase and endoglucanase.

**Table 2:** Comparison of specific activities of the three enzyme preparations on model substrates

Specific activity (U/mg protein)	SP	ST	Celluclast 1.5L
*p*NPGase	0.56 ± 0.02	1.92 ± 0.02	1.43 ± 0.01
CMCase	26.41 ± 1.06	23.53 ± 3.70	24.55 ± 1.01
*p*NPCase	0.21 ± 0.005	0.09 ± 0.002	0.06 ± 0.008
FPA	1.14 ± 0.13	1.26 ± 0.06	1.21 ± 0.04
Xylanase	59.69 ± 4.86	27.40 ± 2.43	6.07 ± 1.01
*p*NPXase	0.29 ± 0.01	1.04 ± 0.01	0.53 ± 0.01

**Fig. 1:**
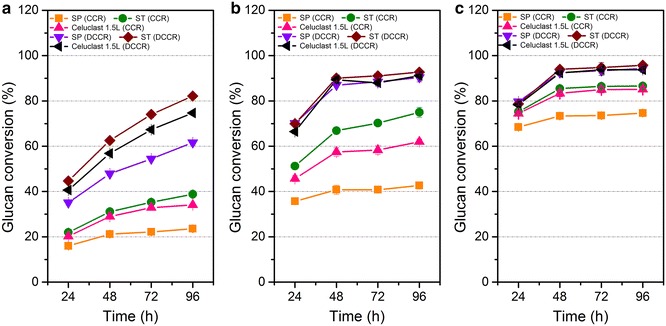
Conversion of glucan during hydrolysis of CCR and DCCR. Enzyme loadings were set at 3.6 mg protein/g glucan (**a**), 20 mg protein/g glucan (**b**), and 100 mg protein/g glucan (**c**), respectively. The values shown are the mean of three replicates and the *error bars* indicate standard deviations from the mean values

To further ascertain the performance of different enzyme preparations on complex lignocellulosic materials, we individually tested glucan conversion rates of SP, ST, and Celluclast 1.5L with the DCCR and CCR substrates, at three different enzyme loadings (3.6, 20, and 100 mg protein/g glucan). The results are shown in Fig. 1. After 96 h of hydrolysis, a significantly higher amount of glucose was released by all the three cellulase preparations from DCCR compared with CCR at each enzyme loadings. At a relatively low enzyme loading of 3.6 mg protein/g glucan, the highest glucan conversion (about 82 %) was achieved by ST enzyme preparation from DCCR at 96 h, 1.34 times as high as that (about 61 %) by SP enzyme preparation (Fig. 1a). At enzyme loadings of 20 and 100 mg protein/g glucan, the three enzyme preparations applied to DCCR (Fig. 1b, c) had similar glucan conversion rates, with a final rate of about 90 % achieved at 48 h.

The glucan conversion decreased distinctly when lignin-containing CCR was employed as a substrate. This was probably due to the hindering of cellulose accessibility by lignin. Interestingly, although the filter paper activities of SP, ST, and Celluclast 1.5L were similar, the latter two gained higher glucose yields from CCR at the same enzyme loadings compared with SP. The data showed that even at high-enzyme loadings, the enzyme systems (especially SP) could not degrade corncob residue completely and thus need to be optimized. Therefore, relatively high-enzyme loadings are used in the following studies. Considering their respective hydrolytic performances on model substrates, we speculated that the reduction in saccharification efficiency of the SP cellulase preparation was caused by deficiencies in some components of the lignocellulolytic enzyme complex.

### Improvement in hydrolytic efficiency of SP by beta-glucosidase supplementation

**Fig. 2:**
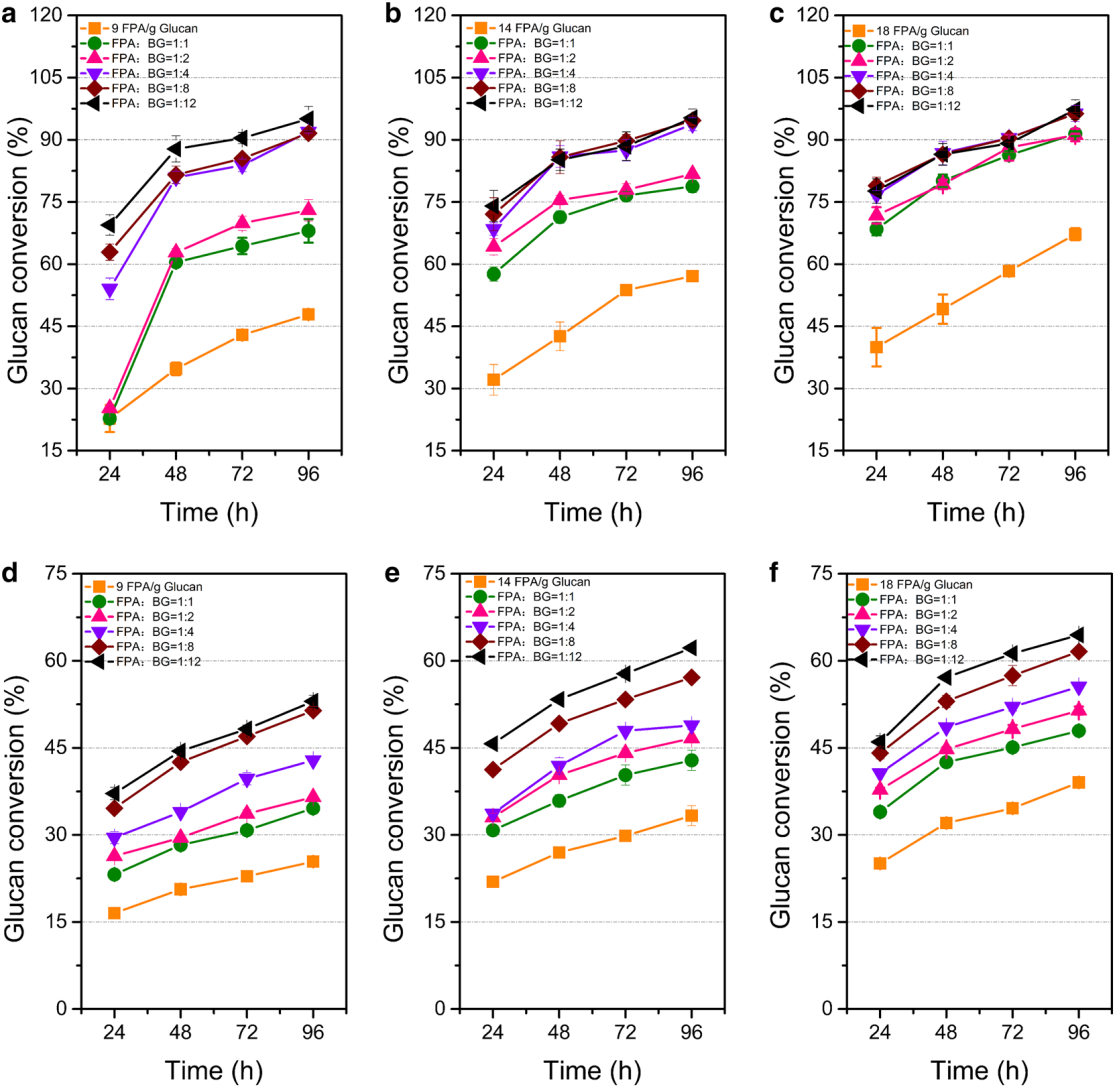
Enhancement of saccharification by BG supplementation. The curves show the glucan conversion rates with DCCR at **a** 9 FPA/g glucan, **b** 14 FPA/g glucan, and **c** 18 FPA/g glucan, and with CCR at **d** 9 FPA/g glucan, **e** 14 FPA/g glucan, and **f** 18 FPA/g glucan. The values shown are the mean of three replicates and the *error bars* indicate standard deviations from the mean values

Previous studies showed that beta-glucosidase (BG) played a significant role by removing cellobiose that inhibits CBHs and EGs [27, 28]. Supplementation of BG to cellulase enzyme mixtures has been shown to improve the overall hydrolysis of pretreated lignocellulosic substrates [29–31]. In addition, the aforementioned analysis in this study showed a specific BG activity of 0.56 U/mg protein for SP, which was obviously lower than that of ST (1.92 U/mg protein). The result suggested that BG in SP might be was insufficient in hydrolyzing biomass substrates. Therefore, various amounts of BG were added to SP to improve hydrolytic efficiency. When a low amount (2.25 U/g glucan) of BG was added to SP, the enzyme loading required for a 92 % glucan conversion from DCCR decreased from 25 filter paper activity (FPA)/g glucan (data not shown) to 9 FPA/g glucan (Fig. 2a). High-performance liquid chromatography (HPLC) analysis revealed undetectable cellobiose levels (see Additional file 1: Figure S1 a–c) when BG was added. This finding suggests that BG relieved the product inhibition of cellulase by cellobiose and promoted glucose yield. However, when the SP enzyme loading increased to 14 and 18 FPA/g glucan, the final glucan conversion rates after BG supplementation were only improved to a small extent compared with that of 9 FPA/g glucan after supplementation (Fig. 2b, c). Hence, a certain composition of enzyme system is necessary to achieve an optimal synergism among CBH, EG, and BG, whereas excessive enzyme loading increases hydrolysis cost but does not evidently improve degradation efficiency.

When CCR was used as substrate, glucan conversion rates reached up to 52, 61, and 65 %, with a high ratio of BG (FPA:BG = 1:12) under cellulase loadings of 9, 14, and 18 FPA/g glucan, respectively (Fig. 2d–f). Cellobiose was undetectable and the xylose content was higher compared with those of DCCR (see Additional file 1: Figure S1 d–f). Although BG addition relieved the cellobiose inhibition, the glucan conversion rate of CCR was still obviously lower than DCCR. The optimal cellulase system on DCCR was not suitable for CCR, which could be caused by the different structures and constituents of the two substrates. The lignin in CCR might block the access of cellulases in SP to cellulose.

### Disappearance of the cellulase components in the supernatants during the process of saccharification

**Fig. 3:**
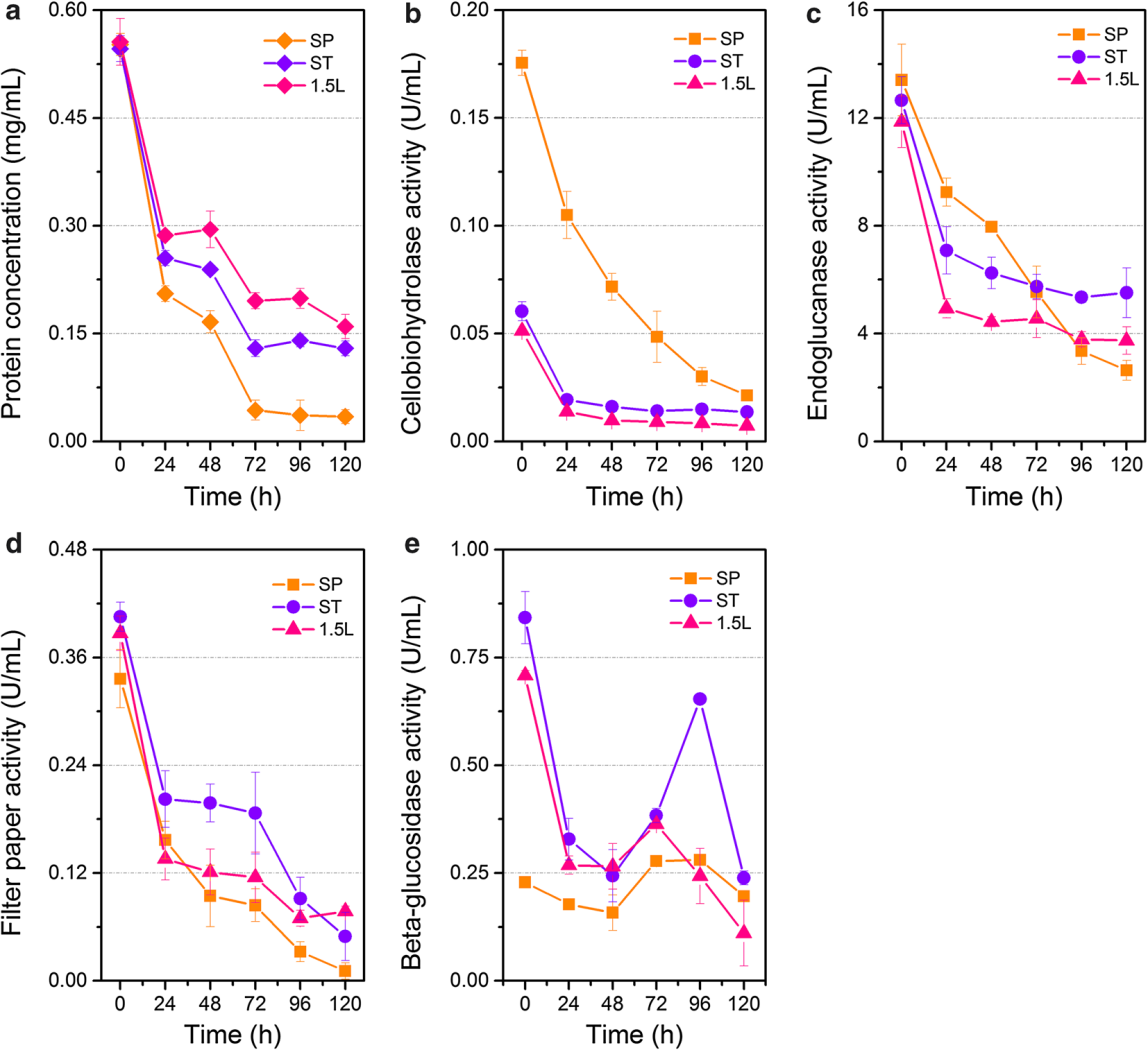
The dynamic changes during saccharification at the enzyme loading of 20 mg protein/g glucan. **a** Protein contents. **b**–**e** The activities of CBH, EG, filter paper, and BG, respectively. The values shown are the mean of three replicates and the *error bars* indicate standard deviations from the mean values

**Fig. 4:**
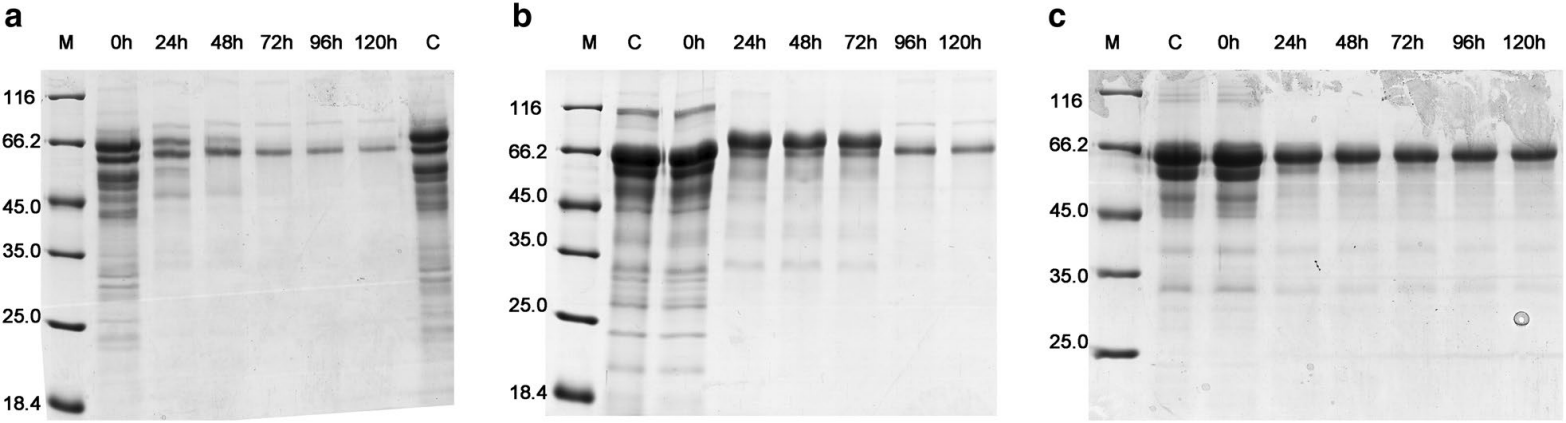
Protein profiles of saccharification supernatants analyzed by SDS-PAGE. **a** SP; **b** ST; **c** Celluclast 1.5L. Enzyme loadings were set at 20 mg protein/g glucan. Samples were collected after saccharification for 0, 24, 48, 72, and 96 h, respectively. Enzyme solution before saccharification was used as control (*lane* *C*)

To further investigate the cause of decreased efficiency of SP in CCR hydrolysis, the dynamic changes of enzymes during the process of saccharification were monitored. The changes in protein content and individual enzyme activities in the supernatants during saccharification when the enzyme dosage was 20 mg protein/g glucan are shown in Fig. 3. A sharp drop in the protein concentration was observed in the supernatants. The protein adsorption on the solid residues of SP was greater than those of ST and Celluclast 1.5L. The adsorption capacity decreased in the order SP > ST > Celluclast 1.5L. The time-course analysis of protein components using sodium dodecyl sulfate–polyacrylamide gel electrophoresis (SDS-PAGE) suggested that the enzyme decreases in the supernatants were due to adsorption rather than protein degradation (Fig. 4). After 24 h, a majority of protein bands were not observed. Unexpectedly, several bands were retained in the supernatants of the three cellulase preparations.

Similar behaviors of individual enzyme activities were also observed during the CCR hydrolysis (Fig. 3b–e). Results show that CBH and EG activities significantly decreased, which may be attributed to the binding to cellulose or lignin. In particular, the BG activities of the three cellulase preparations decreased first, increased subsequently, and then decreased at the end of saccharification. A previous study reported that BG was the enzyme least affected by the lignin because of the absence of cellulose binding module [32]. Hence, the decrease in BG levels in this study was possibly caused by adsorption to insoluble cellulose.

**Fig. 5:**
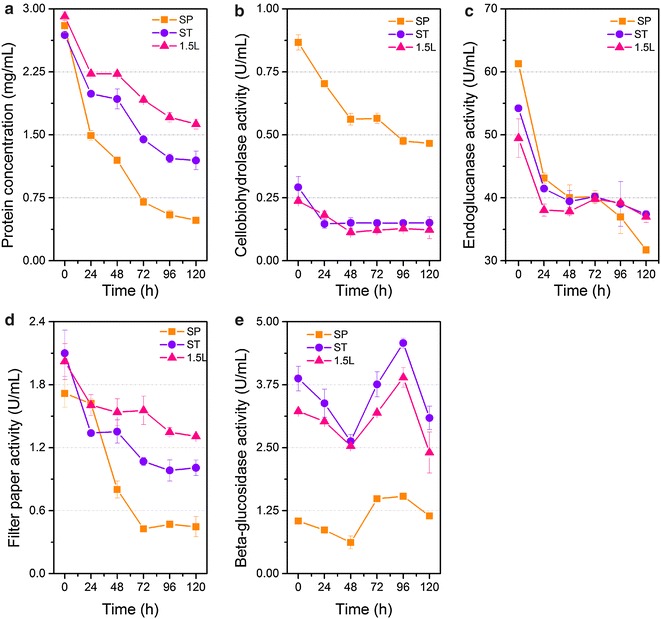
The dynamic changes during saccharification at the enzyme loading of 100 mg protein/g glucan. **a** Protein contents. **b**–**e** The activities of CBH, EG, filter paper, and BG, respectively. The values shown are the mean of three replicates and the *error bars* indicate standard deviations from the mean values

In summary, the SP cellulase preparation was more notably affected by lignin than ST and Celluclast 1.5L, which is not beneficial for biomass degradation. We speculated that the decrease in saccharification efficacy was caused by the adsorption by lignin. To test this hypothesis, enzyme loading was increased to 100 mg protein/g glucan to have a sufficient amount of enzymes during saccharification. Consequently, the glucan conversion rate of CCR by SP at 96 h increased from 40–70 % (Fig. 1). The difference in saccharification between the cellulase preparations became smaller compared with those at lower enzyme dosages. The protein concentrations and filter paper activities of all the three samples decreased gradually (Fig. 5). The SP cellulase preparation displayed remarkably higher CBH and lower BG activities than the other two preparations during the whole saccharification. The results indicate that the excess of CBH activity in SP might not address the obstacles of lignin. Combined with the results of BG supplementation, we suggest that SP possesses other bottleneck(s) in the process of enzymatic hydrolysis in addition to BG insufficiency. This bottleneck could be low specific activity of CBHs on cellulose in spite of high activity on *p*NPC, or the lacking of other enzyme components like beta-xylosidase (Table 2).

### Comparative proteomic analysis

**Fig. 6:**
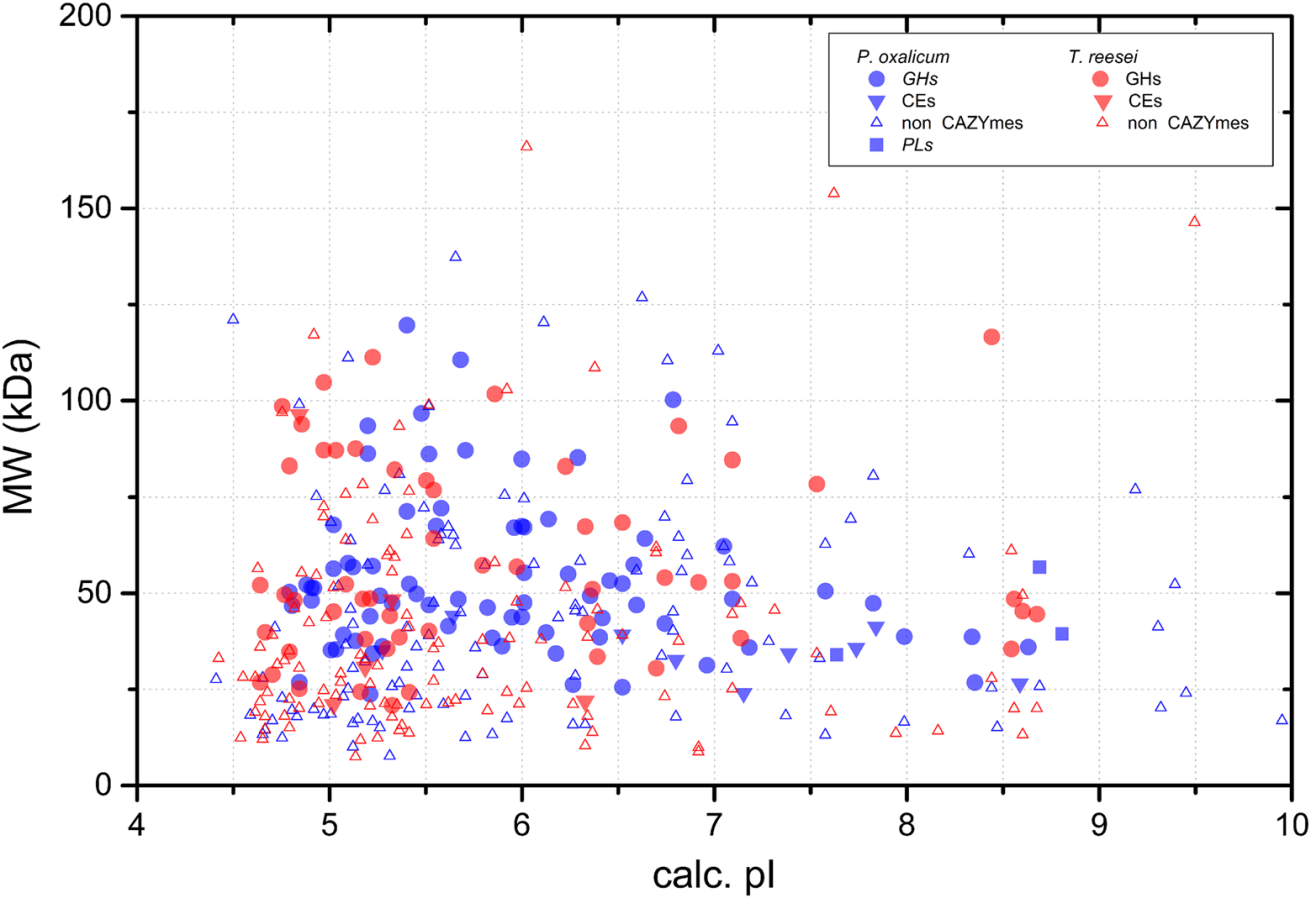
A *plot* of calculated molecular weight against the isoelectric point (pI) of the identified proteins. Proteins annotated with different functions are indicated with *different symbols*

Mass spectrometry-based proteomics study was performed to comprehensively dissect the lignocellulolytic enzyme profile of the enzyme preparations and to further understand the reasons for the difference in hydrolytic ability between SP and ST. The protein groupings based on their peptide-spectrum matches (PSMs) were shown in additional files (see Additional files 2 and 3: Tables S1 and S2). Up to 202 proteins (*P* < 0.05) were unambiguously identified in SP, of which 48 % (97 proteins) were recognized as carbohydrate active enzymes (CAZymes). On the other hand, 173 non-redundant proteins (*P* < 0.05) were identified in the ST cellulase preparation, of which 34 % (59 proteins) were identified as CAZymes. Our results revealed that the molecular weights of all the identified proteins in SP lie in the range of 7.7–137.3 kDa, with the exception of two uncharacterized proteins (240.3 and 863.8 kDa MWs). Meanwhile, 79.2 % of the identified proteins were concentrated within the acidic range. For ST, all the identified proteins lie in the range of 7.5–166 kDa, except for a non-ribosomal peptide synthetase at 539 kDa, and 87.2 % of the identified proteins were concentrated within the acidic range (Fig. 6).

**Fig. 7:**
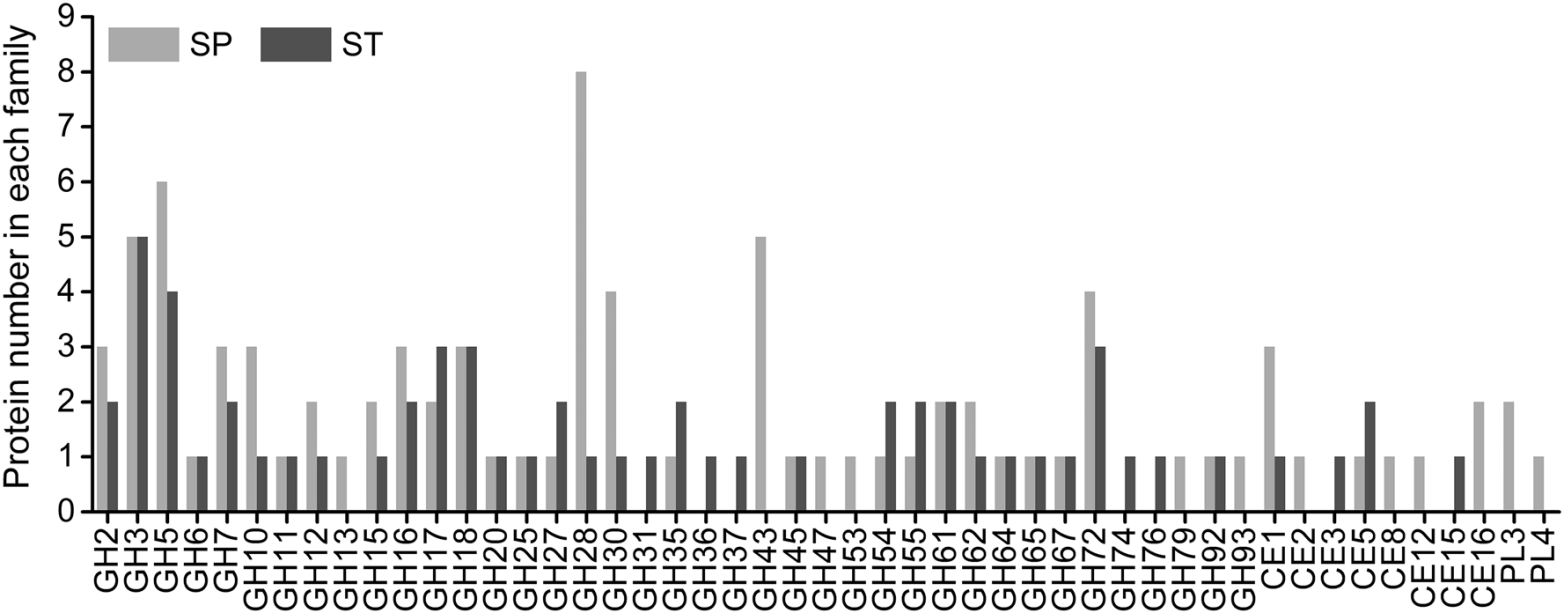
Distribution of GH, CE, and PL family proteins identified in SP and ST

**Fig. 8:**
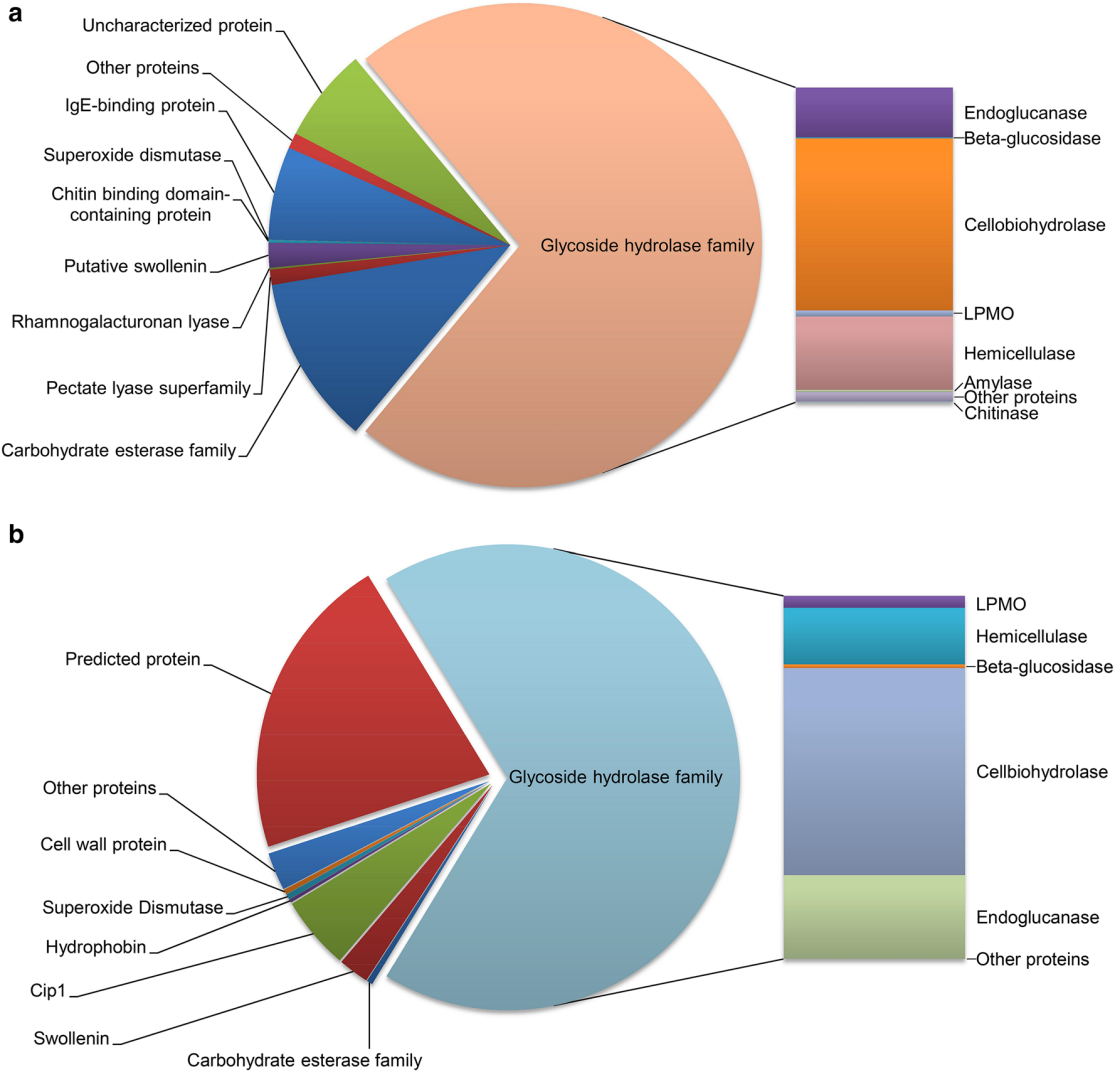
The relative amounts of proteins with different functional classifications identified in SP and ST. Functional predictions of SP (**a**) and ST (**b**) proteins are based on the genome databases for *P. oxallium* and *T. reesei*, respectively. Chitinase and amylase in ST were not shown because of little contents

The number and family distribution of glycoside hydrolases (GHs) were different in two cellulase preparations (Fig. 7). The enzymes of SP and ST can be classified into 35 and 33 GH families, respectively. The SP cellulase preparation was composed of eighteen cellulases, including three CBHs (two CBH Is and one CBH II) and nine EGs. Proteomic data showed that CBH was the most abundant among the lignocellulolytic proteins, followed by hemicellulase, EG, and lytic polysaccharide monooxygenase (LPMO) (Fig. 8a). In addition, SP contained two BGs at low quantities, which accorded its low specific activity of BG. On the other hand, the hemicellulases of SP included six endo-beta-1,4-xylanases, three beta-xylosidases, three acetyl xylan esterase, two feruloyl esterases, and one beta-1,4-mannanase. Acetyl xylan esterases have been reported to promote xylan solubilization by removing acetyl groups from xylan. Feruloyl esterases may facilitate cellulose hydrolysis by hydrolyzing the ester bonds that crosslink lignin and xylan. Also, other proteins including two expansin-like proteins, chitinases, amylases, beta-1,3-glucanosyltransglycosylases, superoxide dismutases [Cu–Zn], and 106 proteins with uncharacterized functions were observed in SP. Notably, acetyl xylan esterase, feruloyl esterases, beta-1,4-mannanase, pectate lyase, pectin esterase, alpha-amylase, polygalacturonase, alpha-L-arabinofuranosidase, exo-beta-1,3-galactanase, alpha-mannosidase, endo-beta-1,4-galactanase, beta-glucuronidase, exo-alpha-L-1,5-arabinanase rhamnogalacturonase, and rhamnogalacturonan lyase were detected exclusively in the proteome of SP. The wide spectrum of CAZymes identified in the SP proteome suggests that *P. oxalicum* produces a suite of enzymes with catalytic versatility for effective lignocellulose degradation. The fungus is then a potentially important source of cellulases and hemicellulases.

Cip1, 4-O-methyl-glucuronoyl methylesterase (Cip2), xyloglucanase, alpha-1,6-mannanases, two hydrophobin, and three cell wall proteins were detected in the ST proteome only (Fig. 8b). The ratios of CBH, EG, BG, LPMO, chitinase, and amylase relative to the total protein in ST were 37, 15, 0.66, 2, 0.017, and 0.011 %, respectively. Meanwhile, the corresponding enzyme ratios in SP were 39, 11, 0.25, 1.37, 0.189, and 0.44 %, respectively. Thus, the amounts of CBH and EG of ST were comparable to those of SP, while the BG and LPMO ratios of ST were higher than those of SP.

### Assessment of the cause of difference in hydrolytic efficiency by adsorption tests

**Fig. 9:**
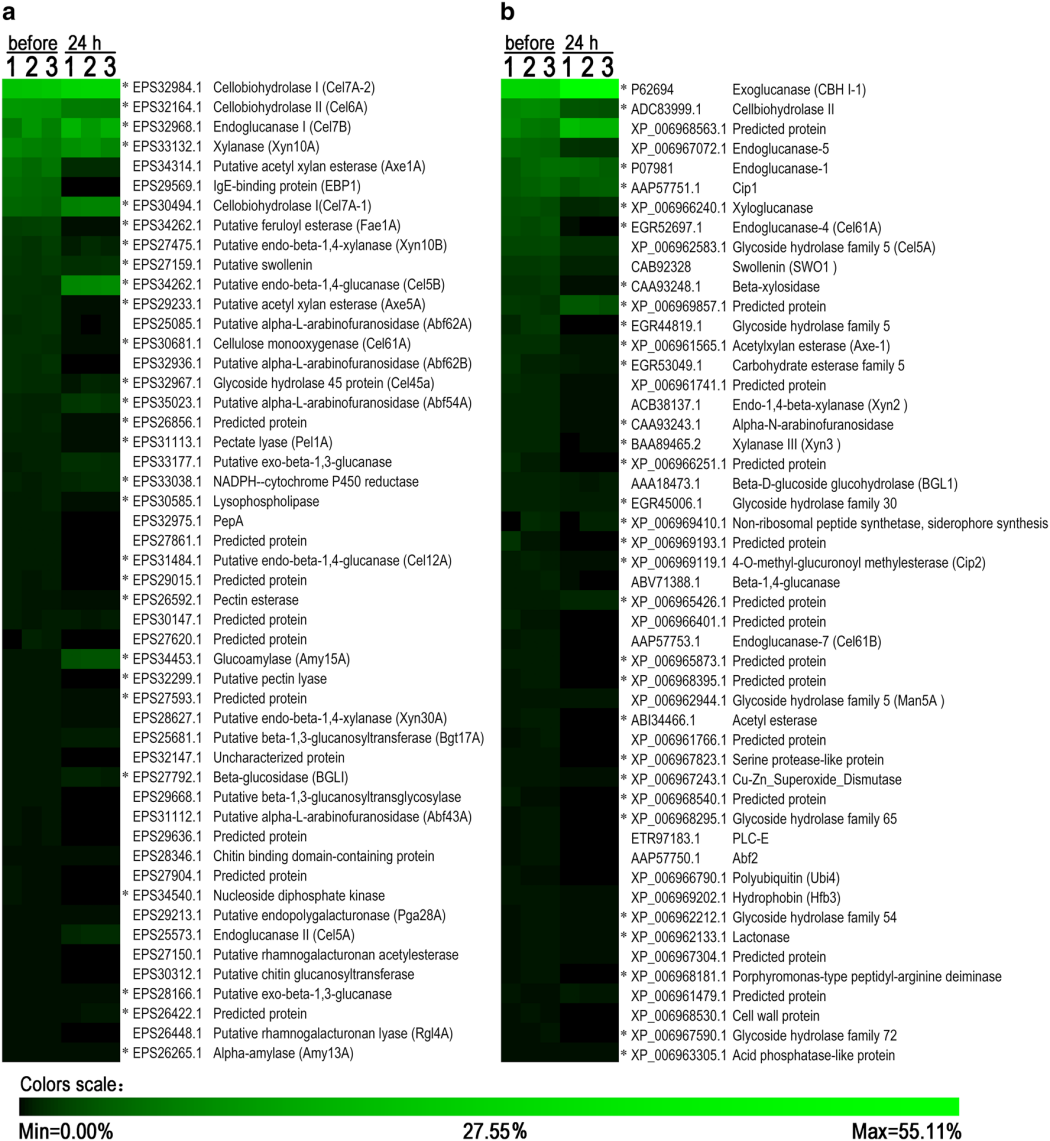
The ratio changes of major components in SP and ST before and after saccharification. **a** SP; **b** ST. For each protein, the ratio in total protein indicated by *color* (in *triplicate*), accession number in GenBank, and functional annotation were shown. A significant difference (*P* < 0.05) was marked with *asterisk*

The saccharification supernatants of SP and ST applied to CCR at enzyme loading of 20 mg protein/g glucan after 24 h were also profiled by proteomic analysis. The relative abundances of the protein components before and 24 h after saccharification were compared. As shown in Fig. 9a, the ratios of swollenin, LPMO (Cel61A), CBH II (Cel6A), and endo-beta-1,4-glucanase (Cel45a) were decreased markedly in the saccharification supernatant of SP. Additionally, IgE-binding protein, alpha-L-arabinofuranosidase, endo-beta-1,4-glucanase (Cel12A), pectin lyase, beta-1,3-glucanosyltransglycosylase, alpha-L-arabinofuranosidase, rhamnogalacturonanacetyl esterase, chitin glucanosyltransferase, rhamnogalacturonan lyase, and xylanase were not detected in the saccharification supernatant. Interestingly, some other EGs (Cel5A, Cel5B, Cel7B), and two CBH Is (Cel7A-1 and Cel7A-2) showed relatively lower extents of decrease considering the dramatic decrease of total protein concentration after saccharification (Fig. 3a). The relative contents of some proteins (e.g., amylase Amy15A) increased, because most of the other proteins bound to substrates.

For ST preparation with a less adsorption of total protein than SP, the relative contents of most proteins also decreased after saccharification (Fig. 9b). Furthermore, 75 % of the total proteins were not detected in the saccharification supernatants. The protein components also showed different adsorptions to substrates according to the calculation of relative ratio changes. Notably, CBH II and LPMO had greater decreases than CBH I and EG I, which is the same with the result in SP study.

Family 1 carbohydrate-binding modules (CBM1 s) in fungal enzymes have been identified to play an important role in the protein adsorption to cellulose and lignin [6, 33]. Many of the identified glycoside hydrolases in fungi possess CBM1. This module increases cellulase concentration on the surface of insoluble substrates and facilitate cellulase catalytic activity. However, CBM1 has been recently shown to be unnecessary or even harmful in the hydrolysis of high-concentration or lignin-containing substrates [34–36]. A total of 20 proteins with CBM1 were found in SP, whereas 11 proteins with CBM1 were found in ST. This finding implies that more enzymes of *P. oxalicum* could be attached to the surface of insoluble lignocellulosic substrates to achieve more efficient degradation. However, the existence of lignin in biomass substrates may also increase the non-productive binding of proteins containing CBM1. In addition, the number of hemicellulases with CBM1 in SP was 10, which was obviously higher than those of ST, with 3 CBM1-containing hemicellulases. The relationship between CBM1 and the stronger adsorption of SP enzymes to lignin is worth to be studied in the future.

## Discussion

*Penicillium oxalicum* can produce large amounts of cellulases and hemicellulases. This fungus has been increasingly investigated because biofuels can be produced from lignocellulosic biomass. Overall, by comparing the hydrolytic efficacies of enzyme preparations on model substrates and natural biomass, we found that the composition of biomass and enzyme system remarkably affect hydrolysis efficiency. This study demonstrated that the presence of lignin negatively influences the saccharification of lignocellulosic biomass by acting as a physical barrier and non-productively adsorbing hydrolytic enzymes. According to the results of enzyme activity analysis and BG supplementation, the BG activity in the SP cellulase preparation was lower than other two cellulase preparations and was insufficient for effective degradation of DCCR and CCR. Undoubtedly, BG was a saccharification bottleneck in SP, and the optimized enzyme system reduced enzyme dosage and hydrolysis cost.

Proteomic analysis revealed that SP contained a wide variety of enzymes acting on main chains and side groups of lignocellulosic materials. Notably, SP contained a group of proteins involved in reducing the recalcitrance of the lignocellulosic matrix via non-hydrolytic action. LPMOs are copper-dependent polysaccharide monooxygenases that oxidatively cleave cellulose. This function is critical in the efficient saccharification of biomass. Intensive studies have shown that LPMO addition can significantly boost the hydrolytic efficiency of cellulase mixtures [37, 38]. LPMO were found in both the SP and ST cellulase preparations. Adsorption tests demonstrated that the LPMOs in the two enzyme preparations were considerably active in hydrolysis, as observed in their dramatically decreased contents in the supernatants. In addition, swollenin has been reported to loosen the plant cell wall matrix through the disruption of hydrogen bonds in polysaccharide networks [18] and improve the enzymatic hydrolysis of crystalline cellulose by cellulases. In this study, swollenin was also found active in binding the substrates.

On the other hand, our data showed that ST highly likely employed strategies different from those of SP to gain access to biomass substrates. We speculated that the unique components xyloglucanase, Cip1, 4-O-methyl-glucuronoyl methylesterase (Cip2), and hydrophobin in ST were capable of promoting substrate degradation by removing the hemicellulose-lignin obstacle. These enzymes have been reported to exhibit some synergistic activity with both LPMO and swollenin [39]. These components may contribute to the high levels of CCR saccharification by ST. Collectively, the LPMOs, swollenin, Cip1, Cip2, and xyloglucanase could serve as desirable targets for the rational construction of an optimal cellulase system.

To the best of our knowledge, this study is the first report that evaluated proteins adsorption by proteomic analysis. Excessive proteins containing CBM1 may be harmful to biomass hydrolysis. The non-productive adsorption of enzyme components on lignin also affected hydrolytic efficiency. Our findings shed light on the complex degradation enzyme system of *P. oxalicum* and highlight the important role of an optimized enzyme system in lignocellulose hydrolysis.

## Conclusions

The cellulolytic ability of *P. oxalicum* was significantly prominent, as demonstrated by its enzyme activity and saccharification efficiency with model substrates. BG supplementation to SP could remarkably reduce enzyme dosage with equal glucose yield. The cellulase system could be optimized by searching for insufficient proteins to help decrease enzyme-related costs in biofuel production. Proteomic method could be applied to reveal the differences in enzyme components between cellulase preparations and the dynamic changes of enzyme systems during saccharification. The proposed method could also be employed to predict targets for the genetic modification of lignocellulolytic enzyme systems.

## Methods

### Cellulase preparation and biomass materials

Commercial cellulase preparations SP and ST in the form of solid powder provided by Sino Biotechnology Co., Ltd. (Baiyin, Gansu, China) produced by *P. oxalicum* JU-A10 (a catabolite repression-resistant mutant strain) and *T. reesei* SN1, respectively, were stored in the laboratory. Celluclast 1.5L was purchased from Sigma–Aldrich (USA).

CCR and DCCR were obtained from Shandong Longlive Bio-Technology Co., Ltd. Before saccharification, CCR and DCCR were milled to achieve small particle sizes ranging from 0.30 to 0.45 mm (diameter) and then stored in sealed bags at room temperature. Analytical methods of CCR and DCCR: Moisture content was determined according to the analytical procedure of National Renewable Energy Laboratory [40]. Acid-soluble lignin (ASL) and acid-insoluble lignin (AIL) were determined according to Chinese Standard Methods [21]. The filtered acid hydrolyzed liquid was neutralized with Ba(OH)_2_ powder and then centrifuged for 15 min, and the glucose and xylose in the supernatant were measured using high-performance liquid chromatography (HPLC) (Shimadzu, Kyoto, Japan) with a refractive index detector (Shimadzu) on an AminexHPX-87P column (Bio-Rad, Hercules, CA, USA) running at a flow rate of 0.5 mL/min at 78 °C, with water as eluent. Glucan and xylan concentrations were calculated using formula (1) and (2), respectively.

1 $$Glucan\,content \,\, (\% ) = \frac{Glucose \, (g) \times 0.9}{Sample\,dry\,weight \, (g)} \,\times\, 100\,\%$$

2 $$Xylan\,content\,(\% ) = \frac{Xylose \, (g) \times 0.88}{Sample\,dry\;weight\,(g)} \times 100 \, \%$$

### Enzyme activity assays

The substrates were purchased from Sigma–Aldrich (USA). The substrates and enzyme preparations were dissolved in 0.05 M sodium citrate buffer (pH 4.8).

FPA, which represented the overall cellulase activity of the samples, was measured using Whatman No. 1 filter paper as the substrate. The reaction mixtures contained 50 mg of filter paper, 1.5 mL of citrate buffer, and 500 µL of the suitably diluted enzyme fraction (protein concentration was 200 µg/mL). These mixtures were then incubated at 50 °C for 60 min. EG and xylanase activities were assayed with CMC-Na and beechwood xylan as the substrates, respectively. The enzyme reactions (protein concentration was 5 µg/mL) were performed in 1 mL of 1 % substrate-containing citrate buffer (pH 4.8) at 50 °C for 30 min. The amount of reducing sugar released was determined via the dinitrosalicylic acid (DNS) method using glucose and xylose as the standard, respectively [41].

CBH activity was assayed with 1.0 mg/mL *p*-nitrophenyl-beta-D-cellobioside (*p*NPC) as a substrate, which contained 1.0 mg/mL D-glucono-1,5-lactone to inhibit the hydrolysis of the substrate by BG. BG activity was assayed with 1.0 mg/mL *p*-nitrophenyl-beta-D-glucoside (*p*NPG) as a substrate. Beta-xylosidase activity was assayed with 1.0 mg/mL *p*-nitrophenylxyloside (*p*NPX) as a substrate. The reactions were carried out using 50 µL of the substrate solution and 100 µL of the enzyme fraction. After incubation was performed at 50 °C for 30 min, the reaction was terminated by adding 150 µL of 10 % (w/v) Na_2_CO_3_ and the released *p*-nitrophenol (*p*NP) was quantified by measuring the absorbance at 420 nm. The inactive enzyme boiled at 100 °C for 10 min was used as the control [42].

One enzyme activity unit (U) was defined as the amount of enzyme that liberates 1 μmol glucose, xylose, or *p*NP per minute under the assay conditions.

### Saccharification of CCR and DCCR

Enzymatic saccharification of CCR and DCCR was carried out in 100 mL Erlenmeyer flasks that contained 5 % (w/v) substrate and enzyme solution in 0.05 M citrate buffer (pH 4.8). A total reaction mixture of 30 mL was incubated at 50 °C with shaking at 150 rpm in a rotary shaker (QB-228). The supernatants at 24, 48, 72, and 96 h of incubation were collected by centrifugation at 10,000 rpm for 10 min, and the glucose concentration was measured using SBA-40C biological sensor analyzer (BISAS, China). The glucan conversion was calculated as follows:

$$Glucan\,conversion\,(\% ) = \frac{Glucose\,yield\,released\,from\,enzyme\,hydrolysis\,(mg)}{Substrate\,weight\,(mg) \times Glucan\,content\,(\% )} \times 0.9 \times 100\, \%$$

All assays were carried out in triplicate, and mean values were calculated.

### Determination of protein concentration

The saccharification supernatants were collected by centrifugation for 10 min at 10,000 rpm and 4 °C. Protein concentrations were determined using a Bradford protein assay kit (Sangon, Shanghai, China) in accordance with the manufacturer’s instructions. Bovine serum albumin was used as standard.

### SDS-PAGE analysis

The same volume samples of 40 μL (concentrations shown in Fig. 3a) were added to 10 μL 5 × loading buffer, boiled for 5 min for denaturation, and loaded in a 12.5 % polyacrylamide gel followed by electrophoresis at 100 V. Coomassie blue G-250 stain reagent was used for staining. The gel pieces were washed with destaining solvent, containing acetic acid, methanol, and water (1:1:8 by volume) until the backgrounds turned clear. Gels were then scanned on a calibrated GS800 scanner (Bio-rad).

### HPLC analysis

In the experiments of beta-glucosidase (*Aspergillus niger* 11101) supplementation, the samples were collected by centrifugation at 10,000 rpm for 10 min at 4 °C and then passed through a 0.25-μm filter. Glucose, cellobiose, and xylose contents were determined by an HPLC system (Shimadzu, Kyoto, Japan) with a refractive index detector (Shimadzu) on an Aminex HPX-87P column (Bio-Rad, Hercules, CA, USA) at a flow rate of 0.5 mL/min at 78 °C. Milli-Q water was adopted as eluent.

### Proteomic analysis

For protein identification, proteomic analysis was performed according to the previously described method [43]. The dissolved cellulase preparations (SP and ST) and saccharification supernatants of CCR after 24 h at enzyme loading of 20 mg protein/g glucan were collected by centrifugation at 10,000 rpm for 10 min at 4 °C. The supernatants at 24 h were chosen because the decreases of enzyme activities have been remarkable at 24 h (Fig. 3), while the concentrations of residual cellulose in the three reactions are more similar to each other than other time points (Fig. 1b). The supernatants were desalted with a 3 kDa molecular cutoff membrane and were precipitated by acetone and trichloroacetic acid (20:1, v/v). The obtained lyophilized protein powders with the same quantities (100 μg) were dissolved in 50 μL denaturation buffer (0.5 M Tris–HCl, 2.75 mM EDTA, and 6 M guanidine-HCl) and subsequently reduced using 30 μL 1 M dithiothreitol at 37 °C for 2 h. The alkylation was performed using iodoacetamide for 1 h away from light, and the samples were desalted and collected using a Microcon YM-10 Centrifugal Filter Unit. The obtained protein samples were digested thoroughly using trypsin (trypsin-protein ratio of 1:25, w/w) for 12 h. The resulting peptide mixtures were lyophilized after desalting with a ZipTip C18 column and then dissolved in double-distilled H_2_O.

LC–MS/MS with a Prominence nano LC system (Shimadzu, Tokyo, Japan) coupled with an LTQ-Orbitrap Velos Pro ETD mass spectrometer (Thermo Scientific, MA, Germany) was used to extract and analyze peptides. The LC system, equipped with a custom-made silica column (75 μm × 15 cm) packed with C18 reversed-phase column (Reprosil-Pur 120 C18-AQ, particle size 3 μm, Dr. Maish, Germany), was used to separate peptides, which were eluted with a stepping gradient of 2 % (v/v) solvent B (0.0–5.0 min), 2–15 % (v/v) solvent B (5.0–25.0 min), 15–40 % solvent B (25.0–55.0 min), 40–98 % (v/v) solvent B (55.0–60.0 min), 98 % solvent B (60.0–70.0 min), 98–2 % (v/v) solvent B (70.0–75.0 min), and 2 % (v/v) solvent B (75.0–90.0 min) at a flow of 300 nL/min. Solvent A was 2.0 % ACN (v/v) in water with 0.1 % (v/v) formic acid and solvent B was 98 % (v/v) ACN in water (v/v) with 0.1 % (v/v) formic acid. The eluted peptides were sprayed into the mass spectrometer via a nanospray ion source with electrospray voltage of 2 kV and transfer capillary temperature of 275 °C. The LTQ-Orbitrap Velos Pro ETD was run in data-dependent acquisition mode with Xcalibur 2.2.0 software (Thermo Scientific). Full-scan MS spectra (from 400 to 1800 m/z) were detected in the Orbitrap with a resolution of 60,000 at 400 m/z. The 10 most intense precursor ions greater than the threshold of 5000 counts in the linear ion trap were selected for MS/MS fragmentation analysis at a normalized collision energy of 35 %. In order to avoid repetitively selecting peptides, dynamic exclusion was used within 60 s. A total of three technical replicates were performed for each sample.

Proteome Discoverer software 1.4 (Thermo Scientific) with the SEQUEST search engine was used for data searches. Liquid chromatography-MS/MS analysis data were identified by searching the *P. oxalicum* and *T. reesei* protein sequence databases downloaded from Uniprot (http://www.uniprot.org). The MS/MS search was done in accordance with the following settings: (1) trypsin was used to digest the proteins allowing two missed cleavages, (2) a precursor mass tolerance of 10 ppm and a fragment mass tolerance of 0.8 Da were set for mass tolerance, and (3) oxidation of methionine was chosen as the dynamic modification and carbamidomethyl of cysteine residues was selected as the fixed modification. Only peptides with at least six amino acid residues showing 95 % certainty (*P* ≤ 0.05) were included in the results. At least two peptides (*P* < 0.05) were needed to be considered for protein identification, and the false discovery rate was set as 1 %.

For the comparison of protein ratios before and after saccharification, statistical analysis was performed using *t* Student two-tail test with the software Microsoft Office 2013 Excel (Microsoft, USA). Differences with *P*  <  0.05 were considered to be significant.

## Additional files

10.1186/s13068-016-0477-2 The saccharification products analyzed by HPLC. (a–c) DCCR was used as substrates, (d–f) CCR was used as substrates. The released sugars during the saccharification were analyzed by HPLC.
10.1186/s13068-016-0477-2 The functional annotations of proteins identified in the proteome of SP. Mass spectrometry-based proteomics study was performed to comprehensively dissect the lignocellulolytic enzyme profile of SP. Accession, Protein name, PSM, Calc. MW, CBM, Calc. pI and CAZy family of identified proteins were shown.
10.1186/s13068-016-0477-2 The functional annotations of proteins identified in the proteome of ST. Mass spectrometry-based proteomics study was performed to comprehensively dissect the lignocellulolytic enzyme profile of ST. Accession, Protein name, PSM, Calc. MW, CBM, Calc. pI and CAZy family of identified proteins were shown.

## Abbreviations

BG: beta-glucosidase
CBH: cellobiohydrolase
EG: endoglucanase
CBM: carbohydrate-binding module
CCR: corncob residue after hemicellulose extraction
DCCR: alkali delignified corncob residue
AIL: acid-insoluble lignin
ASL: acid-soluble lignin
SP: enzyme preparation from *P. oxalicum* JU-A10
ST: enzyme preparation from *T.reesei* SN1
1.5L: Celluclast 1.5L
MW: molecular weight
SDS-PAGE: sodium dodecyl sulfate polyacrylamide gel electrophoresis
*p*NPC: 4-nitrophenyl-beta-D-cellobioside
CMC: carboxymethyl cellulose
FPA: filter paper activity
*p*NPG: 4-nitrophenyl-beta-D-glucopyranoside
*p*NPX: *p*-nitrophenyl-beta-D-xylopyranoside
LPMO: lytic polysaccharide
GH: glycoside hydrolase
CE: carbohydrate esterase
PL: polysaccharide lyase
