# Supplementary figures and images for: Proteomic analysis of the biomass hydrolytic potentials of Penicillium oxalicum lignocellulolytic enzyme system
Source: Biotechnol Biofuels. 2016 Mar 17;9:68. doi: 10.1186/s13068-016-0477-2 (PMC4797192; doi:10.1186/s13068-016-0477-2)

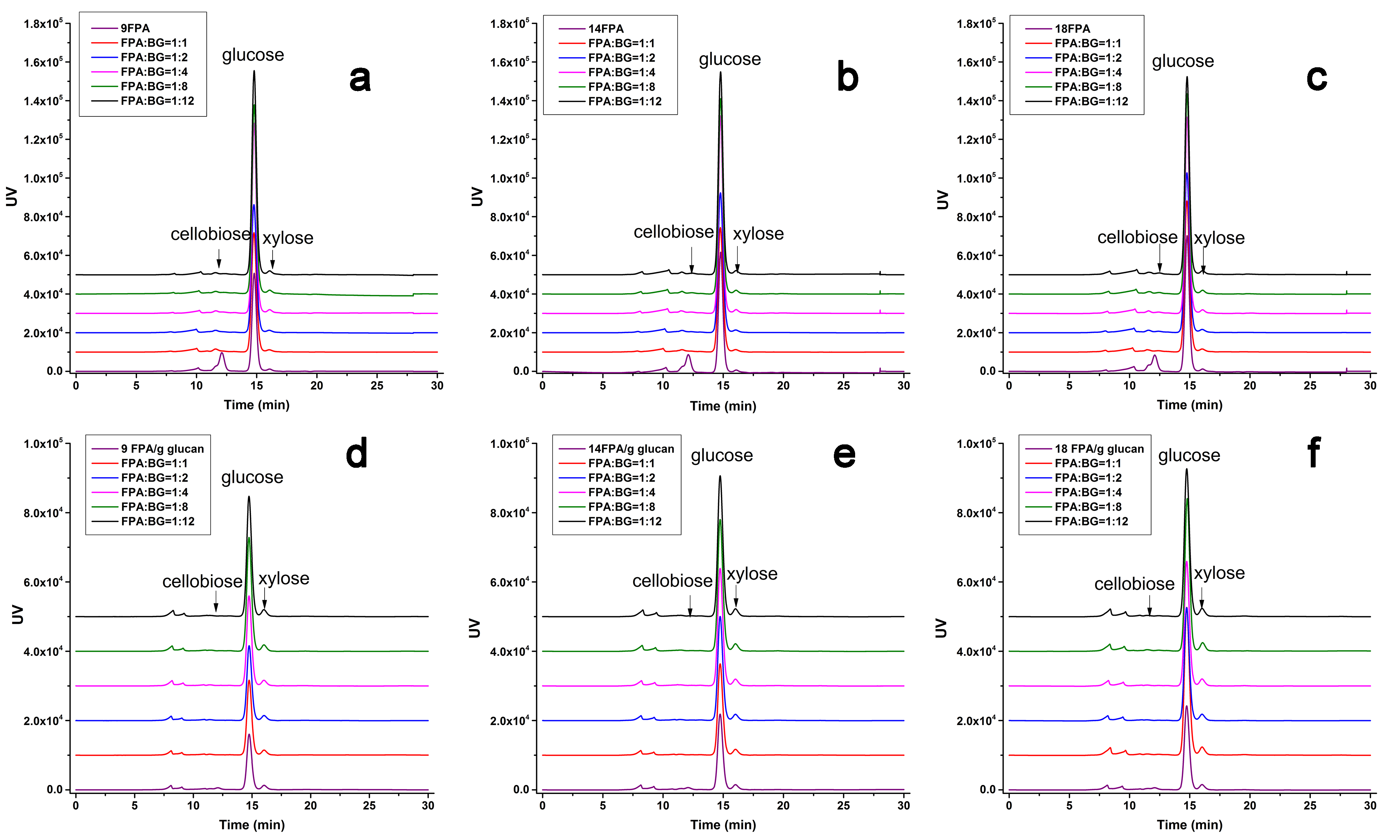

Supplement: Supplementary file 1 — 10.1186/s13068-016-0477-2 The saccharification products analyzed by HPLC. (a–c) DCCR was used as substrates, (d–f) CCR was used as substrates. The released sugars during the saccharification were analyzed by HPLC. [file 13068_2016_477_MOESM1_ESM.tif]
