# Supplementary material for: Proteomic analysis of the biomass hydrolytic potentials of Penicillium oxalicum lignocellulolytic enzyme system
Source: Biotechnol Biofuels. 2016 Mar 17;9:68. doi: 10.1186/s13068-016-0477-2 (PMC4797192; doi:10.1186/s13068-016-0477-2)
Supplement: Supplementary file 2 — 10.1186/s13068-016-0477-2 The functional annotations of proteins identified in the proteome of SP. Mass spectrometry-based proteomics study was performed to comprehensively dissect the lignocellulolytic enzyme profile of SP. Accession, Protein name, PSM, Calc. MW, CBM, Calc. pI and CAZy family of identified proteins were shown. [file 13068_2016_477_MOESM2_ESM.pdf]

Table S1The functional annotations of proteins identified in the proteome of SP

Accession[a]	Protein name	PSMs[b]	Calc. MW[d] [kDa]	Features of CBM[e]	Calc. pI[f]	CAZy family
EPS32984.1	Cellobiohydrolase I (Cel7A-2)	641	56.9	CBM1	5.12	GH7
EPS32164.1	Cellobiohydrolase II (Cel6A)	210.5	48.5	CBM1	5.67	GH6
EPS33132.1	Xylanase (Xyn10A)	185	43.5	CBM1	6.42	GH 10
EPS32968.1	Endoglucanase I (Cel7B)	177.5	49.3	CBM1	5.26	GH7
EPS30494.1	Cellobiohydrolase I(Cel7A-1)	160	48.1		4.91	GH7
EPS29569.1	IgE-binding protein (EBP1)	112	19.6		4.81	
EPS34314.1	Putative[c] acetyl xylan esterase (Axe1A)	104	41.3	CBM1	7.84	CE1
EPS34262.1	Putative[c]endo-beta-1,4-glucanase (Cel5B)	69	44.0	CBM1	5.21	GH5
EPS27159.1	Putative[c]swollenin	56.5	51.9	CBM1	5.05	
EPS34262.1	Putative[c]feruloyl esterase (Fae1A)	55.5	35.7	CBM1	7.74	CE1
EPS27475.1	Putative[c] endo-beta-1,4-xylanase (Xyn10B)	51.5	36.0		8.63	GH10
EPS29233.1	Putative[c] acetyl xylan esterase (Axe5A)	36	24.0		7.15	CE5
EPS28627.1	Putative[c] endo-beta-1,4-xylanase (Xyn30A)	31	52.4		5.41	GH30
EPS25085.1	Putative[c]alpa-L-arabinofuranosidase (Abf62A)	30.5	41.5	CBM1	5.62	GH62
EPS32936.1	Putative[c]alpa-L-arabinofuranosidase (Abf62B)	29.5	35.9		7.18	GH62
EPS31113.1	Pectate lyase (Pel1A)	29	34.0		7.64	PL3
EPS35023.1	Putative[c] alpha-L-arabinofuranosidase (Abf54A)	28.5	52.5	CBM42	6.52	GH54
EPS30681.1	Cellulose monooxygenase (Cel61A)	24.5	26.2		6.27	GH61
EPS34453.1	Glucoamylase (Amy15A)	24.5	67.2	CBM20	6.01	GH15
EPS27792.1	Beta-glucosidase (BGLI)	24	93.4		5.20	GH3
EPS29668.1	Putative[c] beta-1,3-glucanosyltransglycosylase	21	49.8		5.45	GH72
EPS32299.1	Putative[c] pectin lyase	21	39.4		8.81	PL3
EPS29015.1	Predicted protein	18.5	33.1		7.55	
EPS32967.1	Endoglucanase (Cel45A)	18.5	26.9	CBM1	4.84	GH45
EPS30585.1	Lysophospholipase	17.5	68.5		5.01	
EPS31112.1	Putative[c] alpha-L-arabinofuranosidase (Abf43A)	17	49.3	CBM6	6.35	GH43
EPS31484.1	Putative[c] endo-beta-1,4-glucanase (Cel12A)	16	25.5		6.52	GH12
EPS25681.1	Putative[c] beta-1,3-glucanosyltransferase (Bgt17A)	15.5	34.3		5.22	GH17
EPS26856.1	Predicted protein	15	19.9		4.92	
EPS27571.1	Putative[c]alpa-galactosidase (Aga27A)	13.5	55.0	CBM1	6.24	GH27
EPS32625.1	Putative[c]alpa-L-arabinofuranosidase	13	34.3		6.18	GH43
EPS26448.1	Putative[c]rhamnogalacturonanlyase (Rgl4A)	12.5	56.8		8.69	PL4
EPS25573.1	Endoglucanase II (Cel5A)	12	43.9	CBM1	6.00	GH5
EPS33177.1	Putative[c] exo-beta-1,3-glucanase	11.5	85.2		6.29	GH55

EPS26399.1	Predicted protein	11.5	18.4		4.97	
EPS26592.1	Pectin esterase	11	34.3		7.39	CE8
EPS27150.1	Putative[c] rhamnogalacturonanacetyl esterase	11	26.5		8.59	CE12
EPS26265.1	Alpha-amylase (Amy13A)	10.5	67.5	CBM20	5.55	GH13
EPS28506.1	Putative[c] beta-1,3-glucanosyltransglycosylase	10	57.0	CBM43	5.22	GH72
EPS27593.1	Predicted protein	10	18.6		5.01	
EPS35004.1	Endoglucanase (Cel5C)	9.5	69.3	CBM1	6.14	GH5
EPS30243.1	Putative[c] endo-beta-1,4-glucanase (Cel5D)	9.5	35.2		5.01	GH5
EPS31992.1	Putative[c] beta-1,3-glucanosyltransglycosylase	9	57.8	CBM43	5.10	GH72
EPS31445.1	Putative[c] beta-N-acetylhexosaminidase	8.5	36.2		5.27	GH3
EPS32975.1	PepA	8.5	37.5		7.28	
EPS34746.1	Putative[c] beta-xylosidase	8.5	84.8		6	GH3
EPS25084.1	Putative[c] alpha-L-arabinofuranosidase(Abf43B)	8	62.2	CBM1	7.05	GH43
EPS31069.1	Putative[c] beta-1,4-mannanase (Man5A)	8	47.4	CBM1	5.33	GH5
EPS27904.1	Predicted protein	7.5	75.5		5.91	
EPS30147.1	Predicted protein	7.5	43.7		6.19	
EPS28520.1	Putative[c] rhamnogalacturonase	7	47.0		6.60	GH28
EPS34315.1	Putative[c] exo-beta-1,3-galactanase	7	48.5		7.09	GH43
EPS29213.1	Putative[c] endopolygalacturonase (Pga28A)	7	38.4		5.85	GH28
EPS26366.1	Putative[c] acetyl xylan esterase (Axe2A)	7	44.1	CBM1	5.64	CE2
EPS30312.1	Putative[c] chitin glucanosyltransferase	6.5	46.8		4.81	GH16
EPS34540.1	Nucleoside diphosphate kinase	6.5	16.5		7.99	
EPS27857.1	Putative[c] beta-glucuronidase	6.5	71.3		5.40	GH2
EPS27063.1	Putative[c] beta-1,6-glucanase	6.5	51.4		4.91	GH30
EPS25468.1	Putative[c] chitin glucanosyltransferase	6.5	39.2		5.07	GH16
EPS27861.1	Predicted protein	6	26.6		5.36	
EPS28166.1	Putative[c] exo-beta-1,3-glucanase	6	50.3		4.79	GH17
EPS31556.1	Beta-hexosaminidase	5.5	67.0		5.96	GH20
EPS30987.1	Predicted protein	5	36.1		5.45	
EPS28350.1	Putative[c] lysozyme	5	23.7		5.21	GH25
EPS29963.1	Predicted protein	5	32.2		5.19	
EPS28765.1	Putative[c] endo-beta-1,4-glucanase	5	50.6	CBM1	7.58	GH5
EPS29415.1	Predicted protein	4.5	28.5		6.28	
EPS25118.1	Putative[c] beta-xylosidase (Xyl3A)	4.5	87.0		5.71	GH3
EPS33160.1	Putative[c] chitinase (Chi18A)	4.5	46.9		5.52	GH18
EPS31378.1	Predicted protein	4.5	20.0		5.41	
EPS26394.1	Putative[c] guanyl-specific ribonuclease (RNase1)	4.5	13.3		4.65	
EPS26435.1	Putative[c] acid phosphatase	4.5	46.8		6.28	
EPS34254.1	SUN domain-containing protein	4	45.9		5.11	GHNC
EPS27639.1	Putative[c] endo-beta-1,4-galactanase	4	38.6		6.40	GH53
EPS30575.1	Glucoamylase (Amy15B)	3.5	67.5	CBM20	6.00	GH15
EPS30563.1	Putative[c] beta-1,3-glucanase	3.5	46.3		5.82	GH64

EPS28042.1	Predicted protein	3.5	30.8		5.40	
EPS32301.1	Predicted protein	3.5	63.7		5.11	
EPS32147.1	Predicted protein	3.5	15.1		8.47	
EPS26232.1	Putative[c] chitinase	3.5	35.4		5.03	GH18
EPS35021.1	Predicted protein	3	25.4		8.44	
EPS33107.1	Predicted protein	3	18.1		7.37	
EPS34214.1	Predicted protein	3	58.4		6.30	
EPS34435.1	Superoxide dismutase[Cu-Zn]	3	15.8		6.27	
EPS33747.1	Predicted protein	3	30.3		7.06	
EPS31233.1	Predicted protein	3	64.6		6.81	
EPS25816.1	Putative[c] endo-beta-1,4-xylanase (Xyn10C)	3	39.8		6.13	GH10
EPS25083.1	Endo-beta-1,4-xylanase (Xyn30B)	3	55.3	CBM1	6.01	GH30
EPS29403.1	Predicted protein	3	81		5.36	
EPS28309.1	Predicted protein	3	15		5.26	
EPS26858.1	Predicted protein	3	75.2		4.93	
EPS25731.1	Predicted protein	3	12.4		4.75	
EPS34622.1	Predicted protein	3	41.0		4.72	
EPS27203.1	Predicted protein	3	52.8		7.20	
EPS33038.1	NADPH--cytochrome P450 reductase	2.5	76.8		5.29	
EPS32977.1	Putative[c] polygalacturonase	2.5	37.6		5.14	GH28
EPS26344.1	Putative[c] exo-beta-glucosaminidase	2.5	100.2		6.79	GH2
EPS26956.1	Putative[c] exopolygalacturonase	2.5	47.6		6.01	GH28
EPS31044.1	Putative[c] beta-mannosidase	2.5	96.7		5.48	GH2
EPS29948.1	Putative[c] chitinase	2.5	43.7		5.95	GH18
EPS33123.1	Putative[c] alpha-mannosidase	2.5	86.2		5.20	GH92
EPS28346.1	Chitin binding domain-containing protein	2.5	27.6	CBM18	4.41	
EPS26325.1	Cellulose monooxygenase	2	36.2	CBM1	5.90	GH61
EPS29943.1	Predicted protein	2.5	45.2		6.79	
EPS25074.1	Predicted protein	2.5	57.3		5.81	
EPS25661.1	Predicted protein	2.5	62.5		5.66	
EPS34325.1	Predicted protein	2.5	18.0		4.83	
EPS26422.1	Predicted protein	2.5	15.9		6.33	
EPS26316.1	Predicted protein	2.5	16.2		5.12	
EPS28538.1	Predicted protein	2.5	24		9.45	
EPS33120.1	Predicted protein	2	62.1		7.05	
EPS31856.1	Predicted protein	2	39.0		5.52	
EPS31674.1	Putative[c] beta-glucanase	2	67.8		5.02	GH16
EPS30244.1	Putative[c] carbohydrate acetylesterase	1.5	32.6		6.80	CE16
EPS27960.1	Putative[c] beta-glucosidase	1.5	86.1		5.52	GH3
EPS32193.1	Putative[c] endopolygalacturonase	1.5	38.6		8.34	GH28
EPS30977.1	Putative[c] rhamnogalacturonase	1.5	47.4		7.83	GH28
EPS27008.1	Putative[c] beta-glucuronidase	1.5	57.4		6.58	GH79
EPS33001.1	Putative[c] beta-1,3-glucanosyltransglycosylase	1.5	52.2		4.88	GH72
EPS27942.1	Putative[c] endo-beta-1,4-glucanase	1	26.8		8.35	GH12

EPS28272.1	Putative[c] polygalacturonase	1	38.7		7.99	GH28
EPS27158.1	Xylanase (Xyn11A)	1	31.3	CBM1	6.96	GH 11
EPS34369.1	Putative[c] exo-alpha-L-1,5-arabinanase	1	42.1		6.74	GH93
EPS32374.1	Putative[c] beta-xylosidase	1	64.2		6.64	GH43
EPS28903.1	Putative[c] carbohydrate acetylesterase	1	39.2		6.52	CE16
EPS31103.1	Putative[c] endo-beta-1,6-galactanase	1	53.3		6.46	GH30
EPS25145.1	Putative[c] beta-galactosidase	1	110.6		5.68	GH35
EPS32054.1	Putative[c] bifunctional alpha-glucuronidase/N-acetyl beta-glucosaminidase	1	72.1		5.58	GH67
EPS26466.1	Putative[c] alpha, alpha-trehalase	1	119.6		5.40	GH65
EPS33276.1	Putative[c] feruloyl esterase	1	35.1		5.27	CE1
EPS25400.1	Putative[c] alpha-mannosidase	1	56.4		5.02	GH47
EPS34321.1	Putative[c] rhamnogalacturonan alpha-L-rhamnopyranohydrolase	1	51.4		4.92	GH28
EPS31183.1	Ecm33 domain-containing protein	1	41.9		5.12	GHNC
EPS27620.1	Predicted protein	2	41.3		9.31	
EPS26013.1	Predicted protein	2	58.2		7.08	
EPS32842.1	Predicted protein	2	59.8		6.86	
EPS34586.1	Predicted protein	2	55.9		6.6	
EPS29556.1	Predicted protein	2	45.1		6.32	
EPS33808.1	Predicted protein	2	57.5		6.06	
EPS28528.1	Dioxygenase(DO1)	2	41.1		5.40	
EPS31173.1	Predicted protein	2	16.6		5.22	
EPS34994.1	Predicted protein	2	22.8		4.75	
EPS26410.1	Predicted protein	2	16.9		4.70	
EPS34000.1	Predicted protein	2	14.4		4.67	
EPS25161.1	Predicted protein	2	28.0		4.65	
EPS28394.1	Predicted protein	1.5	33.7		6.73	
EPS29407.1	Predicted protein	1.5	57.4		5.20	
EPS32974.1	Predicted protein	1.5	17.2		5.15	
EPS31720.1	Predicted protein	1.5	23.1		5.07	
EPS32493.1	Predicted protein	1.5	18.4		4.59	
EPS30476.1	Predicted protein	1.5	120.3		6.11	
EPS33139.1	Predicted protein	1.5	64.0		5.57	
EPS31855.1	Predicted protein	1	30.5		5.12	
EPS27582.1	Predicted protein	1	19.6		11.88	
EPS30584.1	Predicted protein	1	16.9		9.95	
EPS27064.1	Predicted protein	1	52.3		9.39	
EPS33664.1	Predicted protein	1	20.2		9.32	
EPS34574.1	Predicted protein	1	77.0		9.19	
EPS33311.1	Predicted protein	1	25.8		8.69	
EPS25698.1	Predicted protein	1	240.3		8.40	
EPS25620.1	Predicted protein	1	60.2		8.32	

EPS30282.1	Predicted protein	1	80.5	7.83
EPS32783.1	Predicted protein	1	69.3	7.71
EPS30149.1	Predicted protein	1	62.8	7.58
EPS31978.1	DNA-directed RNA polymerase subunit	1	13.1	7.58
EPS33947.1	Predicted protein	1	94.5	7.09
EPS27500.1	Predicted protein	1	113.0	7.02
EPS29636.1	Predicted protein	1	79.4	6.86
EPS30277.1	Serine/threonine-protein phosphatase	1	55.7	6.83
EPS32828.1	Predicted protein	1	17.9	6.80
EPS32966.1	Predicted protein	1	40.2	6.79
EPS28218.1	Predicted protein	1	110.5	6.76
EPS31812.1	Predicted protein	1	69.8	6.74
EPS33257.1	Predicted protein	1	126.8	6.62
EPS34941.1	Predicted protein	1	45.5	6.28
EPS27188.1	Predicted protein	1	863.8	6.19
EPS27845.1	Predicted protein	1	74.6	6.01
EPS25569.1	Predicted protein	1	17.4	5.92
EPS30898.1	Predicted protein	1	13.3	5.85
EPS32957.1	Predicted protein	1	28.9	5.80
EPS25494.1	Predicted protein	1	35.8	5.76
EPS34730.1	Predicted protein	1	23.3	5.71
EPS32391.1	Predicted protein	1	12.5	5.71
EPS26774.1	Predicted protein	1	45.0	5.68
EPS34733.1	Predicted protein	1	137.3	5.66
EPS26417.1	Predicted protein	1	65.1	5.64
EPS34349.1	Predicted protein	1	67.3	5.62
EPS31676.1	Predicted protein	1	21.1	5.59
EPS25504.1	Predicted protein	1	65.3	5.58
EPS34668.1	Predicted protein	1	30.9	5.57
EPS30550.1	Predicted protein	1	47.5	5.54
EPS30528.1	Predicted protein	1	98.5	5.52
EPS29716.1	Predicted protein	1	72.2	5.49
EPS28491.1	Predicted protein	1	23.4	5.45
EPS27910.1	Predicted protein	1	25.8	5.33
EPS28924.1	Predicted protein	1	7.7	5.31
EPS26338.1	Predicted protein	1	10.1	5.12
EPS32017.1	Predicted protein	1	111.2	5.10
EPS33669.1	Predicted protein	1	25.1	5.10
EPS34012.1	Predicted protein	1	36.6	5.08
EPS30132.1	Predicted protein	1	99.0	4.84
EPS33448.1	Predicted protein	1	121	4.5

[a]Accession numbers from NCBI database.

[b]PSM: peptide-spectrum match. The values of PSMs shown are the mean of three replicates.

[c]Putative protein refers to protein without experimental data in support of the predicted function.

[d]Calc. MW refers to predictedmolecular weight according to the sequence.

[e]CBM refers to carbohydrate binding module.

[f]Calc. pI refers to predicted isoelectric point according to the sequence.
