# Supplementary material for: Proteomic analysis of the biomass hydrolytic potentials of Penicillium oxalicum lignocellulolytic enzyme system
Source: Biotechnol Biofuels. 2016 Mar 17;9:68. doi: 10.1186/s13068-016-0477-2 (PMC4797192; doi:10.1186/s13068-016-0477-2)
Supplement: Supplementary file 3 — 10.1186/s13068-016-0477-2 The functional annotations of proteins identified in the proteome of ST. Mass spectrometry-based proteomics study was performed to comprehensively dissect the lignocellulolytic enzyme profile of ST. Accession, Protein name, PSM, Calc. MW, CBM, Calc. pI and CAZy family of identified proteins were shown. [file 13068_2016_477_MOESM3_ESM.pdf]

Table S2 The functional annotations of proteins identified in the proteome of ST

Accession[a]	Protein name	PSMs[b]	Calc. MW[d] [kDa]	Features of CBM[e]	Calc. pI[f]	CAZy family
P62694	Exoglucanase 1	742.5	54.0	CBM1	4.81	GH7
XP_006966240.1	Xyloglucanase	236	87.1	CBM1	5.78	GH74
ADC83999.1	Cellbiohydrolase II	218.5	49.6	CBM1	5.34	GH6
XP_006962583.1	Glycoside hydrolase family 5 (Cel5A)	192	44.1	CBM1	5.22	GH5
P07981	Endoglucanase（EG-1）	134.5	48.2	CBM1	4.94	GH7
CAA93248.1	Beta-xylosidase	98	87.1		5.78	GH3
AAA18473.1	Beta-D-glucosideglucohydrolase（BGL1）	95	78.4		6.86	GH3
AAP57751.1	Cip1	68.5	32.9	CBM1	5.19	
EGR44819.1	Glycoside hydrolase family 5	65.5	52.8		6.07	GH5
EGR45006.1	Glycoside hydrolase family 30	50	48.5		7.40	GH30
Q9P8D0	Swollenin (SWO1)	48.5	51.5	CBM1	5.02	
CAA93243.1	Alpha-N-arabinofuranosidase	48.5	51.1		6.52	GH54
XP_006968563.1	Predicted protein	48	37.9		6.10	
AAP57753.1	Endoglucanase-7 (Cel61B)	36.5	26.8		7.72	GH61
BAA89465.2	Xylanase III (Xyn3)	35	38.1		7.44	GH10
XP_006968395.1	Predicted protein	35	93.3		5.36	
XP_006968295.1	Glycoside hydrolase family 65	35	116.6		6.90	GH65
XP_006969857.1	Predicted protein	34.5	9.9		6.92	
XP_006969100.1	Alpha-glucuronidase	32	93.4	CBM6，CBM35	6.90	GH67
XP_006968181.1	Porphyromonas-type peptidyl-arginine deiminase	31.5	39.2		4.70	
XP_006967823.1	Serine protease-like protein	30	58.0		5.86	
ACB38137.1	Endo-1,4-beta-xylanase (Fragment) (Xyn2)	29	20.8		8.76	GH11
XP_006967237.1	Predicted protein	29	72.5		4.97	
XP_006969119.1	4-O-methyl-glucuronoyl methylesterase (Cip2)	28.5	48.3	CBM1	7.31	CE15
XP_006967590.1	Glycoside hydrolase family 72	26	57.3		4.89	GH72
XP_006967539.1	Transaldolase	24	35.6		5.54	
XP_006961565.1	Acetylxylan esterase (Axe1)	21.5	30.7	CBM1	5.97	CE1
XP_006963305.1	Acid phosphatase-like protein	21.5	47.8		5.97	
XP_006967072.1	Endoglucanase-5	20	24.4	CBM1	4.42	GH45
XP_006967304.1	Predicted protein	19	29.1		5.06	
XP_006961741.1	Predicted protein	19	20.9		5.35	
XP_006966251.1	Predicted protein	19	13.2		8.60	
XP_006961766.1	Predicted protein	19	21.5		5.62	
XP_006969552.1	Glycoside hydrolase family 31	18.5	98.5		6.76	GH31
XP_006962944.1	Glycoside hydrolase family 5 (Man5A)	18	40.1		5.38	GH5
XP_006967243.1	Cu-Zn_Superoxide_Dismutase	18	20.0		8.68	

XP_006968530.1	Cell wall protein	18	41.2		5.41	
XP_006965873.1	Predicted protein	17	20.0		8.56	
XP_006967558.1	Beta-galactosidase (Bga1)	17	111.3		6.84	GH35
ABV71388.1	Beta-1,4-glucanase	16.5	25.1		7.27	GH12
ABI34466.1	Acetyl esterase	16.5	39.1		6.52	
XP_006965576.1	Predicted protein	16.5	47.4		7.14	
AAP57750.1	Abf2	15	34.8		6.93	GH62
XP_006969947.1	Predicted protein	13.5	117.1		4.92	
XP_006967805.1	Alpha, alpha-trehalase	13	76.9		6.76	GH37
XP_006962133.1	Lactonase	12.5	38.6		6.34	
XP_006966790.1	Polyubiquitin(Ubi4)	11.5	34.3		7.53	
XP_006969985.1	Predicted protein	11.5	51.6		6.23	
XP_006969859.1	Phospholipase-like protein	11.5	70.0		4.97	
XP_006962212.1	Glycoside hydrolase family 54	11	53.1		6.11	GH54
XP_006965281.1	Glycoside hydrolase family 3 (Cel3B)	10.5	93.9		6.09	GH3
XP_006967444.1	Cell wall protein	10	22.4		4.79	
ETR97183.1	PLC-E	10	69.2		5.22	
XP_006967077.1	Predicted protein	10	38.3		5.94	
XP_006964971.1	Predicted protein	10	42.4		4.89	
XP_006967828.1	Glycoside hydrolase family 17 (Fragment)	10	35.6		5.19	GH17
XP_006969845.1	Glycoside hydrolase family 55	10	79.3		6.35	GH55
XP_006963461.1	Aldose-1-epimerase	10	44.5		7.09	
XP_006968466.1	Glycoside hydrolase family 72	10	48.6		5.19	GH72
XP_006968306.1	Aminopeptidase	9.5	55.3		4.86	
XP_006968734.1	Predicted protein	9	153.8		7.62	
XP_006969193.1	Predicted protein	8.5	15.0		4.79	
XP_006961551.1	Predicted protein	8.5	24.3		5.92	
XP_006969396.1	Predicted protein	8.5	25.4		6.02	
XP_006965675.1	Predicted protein	8	43.7		4.98	
XP_006965943.1	Beta-1,3-endoglucanase (Fragment)	8	42.2		4.67	GH17
EGR53049.1	Carbohydrate esterase family 5	8	21.9		6.64	CE5
EGR52697.1	Endoglucanase-4 (Cel61A)	8	35.5	CBM1	5.67	GH61
XP_006969380.1	Amidase	8	59.8		5.30	
XP_006962702.1	Glycoside hydrolase family 27	8	48.5		5.30	GH27
XP_006962122.1	Predicted protein	7.5	11.9		5.16	
XP_006968981.1	Predicted protein	7.5	76.5		5.41	
XP_006964451.1	Predicted protein	7	24.7		4.97	
XP_006966396.1	Protein disulfide isomerase	7	54.7		4.93	
XP_006964241.1	Predicted protein	7	14.3		5.36	
XP_006965432.1	Glycoside hydrolase family 55	7	83.0		6.25	GH55
XP_006962481.1	Predicted protein	7	22.2		5.66	
XP_006968421.1	Non-Catalytic module family expansin	6.5	30.6		4.84	
XP_006966401.1	Predicted protein	6.5	19.2		7.61	
XP_006968342.1	Predicted protein	6.5	45.6		7.31	

XP_006965040.1	Predicted protein	6.5	18.0	6.34	
XP_006963063.1	Beta-hexosaminidase	6.5	64.2	6.28	GH20
XP_006968452.1	Glycoside hydrolase family 76	6.5	45.3	5.30	GH76
XP_006968448.1	Glycoside hydrolase family 5	6	52.3	6.44	GH5
Q92451	Alpha-galactosidase 3 (Aga3)	6	68.4	6.62	GH27
XP_006969287.1	Glycoside hydrolase family 2	6	101.7	5.69	GH2
XP_006969319.1	Alpha-galactosidase	6	82.0	5.95	GH36
XP_006966823.1	Ceramidase family protein	6	78.3	5.17	
XP_006962856.1	Predicted protein	6	60.5	6.70	
XP_006968497.1	Proteinase inhibitor	5.5	96.9	4.75	
XP_006969165.1	Glycoside hydrolase family 2	5	104.7	4.82	GH2
XP_006969202.1	Hydrophobin (Hfb3)	5	10.4	6.33	
XP_006962005.1	Predicted protein	5	14.2	8.16	
XP_006967420.1	Predicted protein	5	31.5	4.73	
XP_006961085.1	Axe2	5	30.5	4.73	GH35
XP_006969479.1	Predicted protein	5	59.4	5.34	
XP_006965325.1	Triosephosphateisomerase	5	27.2	5.54	
XP_006965572.1	Aldose-1-epimerase	5	34.0	5.16	
XP_006964163.1	Predicted protein	4.5	31.2	5.25	
XP_006966283.1	Predicted protein	4.5	21.4	5.29	
XP_006961170.1	Predicted protein	4	19.5	5.82	
XP_006961351.1	Glycoside hydrolase family 28	4	38.3	6.38	GH28
XP_006965622.1	Predicted protein	4	26.9	5.06	
XP_006962814.1	Predicted protein	4	12.1	4.65	
XP_006969909.1	Predicted protein	4	65.3	5.40	
XP_006969309.1	Thioredoxin	4	13.9	6.37	
XP_006966596.1	Predicted protein	4	13.6	7.94	
XP_006964775.1	Predicted protein	4	75.8	5.08	
XP_006963375.1	Glycoside hydrolase family 3	4	84.6	5.54	GH3
XP_006962614.1	Aminopeptidase	4	98.8	5.52	
XP_006968279.1	Predicted protein	3.5	20.1	4.84	
XP_006965256.1	Predicted protein	3.5	12.4	5.25	
XP_006963906.1	Serine carboxypeptidase	3.5	60.9	5.31	
XP_006962048.1	Hydrophobin-2 (Hfb2)	3.5	8.8	6.92	
XP_006962043.1	Predicted protein	3.5	19.1	4.61	
XP_006966740.1	Predicted protein	3.5	20.7	5.21	
XP_006967922.1	Predicted protein	3.5	44.3	5.40	
XP_006962073.1	Predicted protein	3.5	17.8	5.33	
CAL90884.1	Tyrosinase 2	3.5	61.1	8.54	
ADQ89788.1	Chitinase 18-5 (Fragment)	3.5	28.8	5.07	GH18
XP_006965426.1	Predicted protein	3	12.4	4.54	
XP_006962977.1	Predicted protein	3	37.1	5.57	
XP_006965569.1	Predicted protein	3	24.2	4.68	
XP_006964812.1	Predicted protein	3	13.7	5.41	

XP_006967320.1	Putative[c] uncharacterized[d] protein	3	108.5		6.38	
XP_006966365.1	Predicted protein	3	21.1		6.27	
XP_006966294.1	Glycoside hydrolase family 17 (Fragment)	3	56.9		4.86	GH17
XP_006967219.1	Glycoside hydrolase family 92	3	87.5		5.48	GH92
XP_006967591.1	Glucose oxidase	3	63.9		5.08	
XP_006969475.1	Carbohydrate esterase family 3 (Fragment)	3	96.3		5.33	CE3
XP_006969714.1	Glycoside hydrolase family 64	2.5	39.8		4.73	GH64
XP_006968519.1	Predicted protein	2.5	36.0		4.64	
Q6WGJ1	HEX1	2.5	25.2		7.09	
XP_006962788.1	Predicted protein	2.5	35.1		4.79	
EGR53049.1	Carbohydrate esterase family 5	2.5	21.0		4.45	CE5
XP_006968547.1	Nuclear movement protein	2.5	21.0		5.50	
XP_006965484.1	Glycoside hydrolase family 72	2.5	52.1		5.03	GH72
XP_006961280.1	Predicted protein	2	28.1		4.61	
XP_006969938.1	Predicted protein	2	56.5		4.63	
XP_006962411.1	Predicted protein	2	21.8		4.64	
XP_006968695.1	Cell wall protein	2	32.5		4.77	
XP_006968559.1	Predicted protein	2	27.9		8.44	
XP_006963641.1	Glycoside hydrolase	2	45.2		4.83	GH16
XP_006966901.1	Predicted protein	2	14.6		4.67	
AAP57760.1	Cel3E	2	83.0		6.49	GH3
XP_006965688.1	Predicted protein	2	37.6		6.81	
XP_006965901.1	Predicted protein	1.5	28.2		4.55	
XP_006962276.1	Glycoside hydrolase family 25	1.5	24.2		6.37	GH25
XP_006960925.1	Glucoamylase	1.5	67.3	CBM20	5.30	GH15
XP_006969410.1	Non-ribosomal peptide synthetase, siderophore synthesis	1.5	539.0		5.33	
XP_006962014.1	Predicted protein	1.5	46.1		4.82	
XP_006961999.1	Predicted protein	1.5	23.2		6.74	
XP_006968640.1	Predicted protein	1.5	28.9		5.80	
XP_006962472.1	Predicted protein	1.5	26.4		5.21	
ETR98380.1	Chitinase 18-7	1.5	44.5		5.38	GH18
XP_006969005.1	Predicted protein	1.5	23.4		5.03	
XP_006965235.1	Predicted protein	1.5	49.5		8.60	
XP_006963738.1	Glycoside hydrolase family 18	1.5	38.5		5.12	GH18
XP_006963475.1	Predicted protein	1.5	7.5		5.14	
XP_006967517.1	Predicted protein	1.5	102.9		5.92	
XP_006961757.1	Predicted protein	1	21.3		4.94	
XP_006961479.1	Predicted protein	1	18.1		4.77	
XP_006966818.1	Predicted protein	1	55.6		5.33	
XP_006966460.1	Putative[c] uncharacterized[d] protein	1	17.9		4.67	
XP_006968869.1	Predicted protein	1	45.7		6.39	
XP_006968540.1	Putative[c] uncharacterized[d] protein	1	146.3		9.50	
XP_006967842.1	Isocitratelyase	1	61.9		6.70	

XP_006961564.1	Predicted protein	1	15.6	5.38	
XP_006963585.1	Predicted protein	1	166.0	6.02	
XP_006962350.1	Glycoside hydrolase family 16 (Fragment)	1	33.5	4.74	GH16
XP_006964884.1	Predicted protein	1	33.0	4.42	
XP_006969370.1	Predicted protein	1	21.2	5.99	
XP_006965932.1	Predicted protein Autophagy-related protein	1	37.8	5.80	

[a]Accession numbers from NCBI database.

[b]PSM: peptide-spectrum match. The values of PSMs shown are the mean of three replicates.

[c]Putative protein refers to protein without experimental data in support of the predicted function.

[d]Calc. MW refers to predictedmolecular weight according to the sequence.

[e]CBM refers to carbohydrate binding module.

[f]Calc. pI refers to predicted isoelectric point according to the sequence.
